# High-Density Lipoprotein Subfractions and Cholesterol Efflux Capacity Are Not Affected by Supervised Exercise but Are Associated with Baseline Interleukin-6 in Patients with Peripheral Artery Disease

**DOI:** 10.3389/fcvm.2017.00009

**Published:** 2017-03-02

**Authors:** Mazen S. Albaghdadi, Zheng Wang, Ying Gao, R. Kannan Mutharasan, John Wilkins

**Affiliations:** ^1^Department of Medicine, Division of Cardiology, Northwestern University Feinberg School of Medicine, Chicago, IL, USA; ^2^Department of Surgery, Division of Vascular Surgery, Northwestern University Feinberg School of Medicine, Chicago, IL, USA; ^3^Department of Preventive Medicine, Northwestern University Feinberg School of Medicine, Chicago, IL, USA; ^4^Department of Preventive Medicine, Division of Cardiology, Northwestern University Feinberg School of Medicine, Chicago, IL, USA; ^5^Department of Medicine, Division of Cardiology, Northwestern University Feinberg School of Medicine, Chicago, IL, USA

**Keywords:** exercise, peripheral artery disease, high-density lipoprotein, HDL efflux capacity, HDL subfractions

## Abstract

**Objective:**

To quantify the association between high-density lipoprotein (HDL) subfractions, efflux capacity, and inflammatory markers at baseline and the effect of supervised exercise on these HDL parameters in patients with peripheral artery disease (PAD).

**Methods:**

The study to improve leg circulation (SILC) was a randomized trial of supervised treadmill exercise, leg resistance training, or control in individuals with PAD. In a *post hoc* cross-sectional analysis, we quantified the associations between baseline HDL subfraction concentrations (HDL2 and HDL3), HDL-C efflux capacity, and inflammatory markers [C-reactive protein (CRP) and interleukin-6 (IL-6)]. We then examined the effect of supervised exercise on changes in these lipoprotein parameters and inflammatory markers in 88 patients from SILC.

**Results:**

Baseline HDL-C efflux capacity was associated with baseline concentrations of HDL2 (β = 0.008, *p* = 0.0106), HDL3 (β = 0.013, *p* < 0.0001), and IL-6 (β = −0.019, *p* = 0.03). Baseline HDL3 concentration was inversely associated with IL-6 concentration (β = −0.99, *p* = 0.008). Compared to control, changes in HDL2, HDL3, normalized HDL-C efflux capacity, CRP, or IL-6 were not significantly different at 6 months following the structured exercise intervention.

**Conclusion:**

HDL efflux and HDL3 were inversely associated with IL-6 in PAD patients. Structured exercise was not associated with changes in HDL subfractions, HDL-C efflux capacity, CRP, and IL-6 in PAD patients. Our preliminary findings support the theory that inflammation may adversely affect HDL structure and function; however, further studies are needed to evaluate these findings.

## Introduction

Exercise has been observed to alter high-density lipoprotein (HDL) metrics associated with the risk of cardiovascular disease (CVD), including increased HDL subfractions (1) in individuals without known CVD and increased HDL-C efflux capacity in observational studies of athletes (2, 3). Patients with peripheral artery disease (PAD) are at high risk for CVD events, including limb threatening and acute systemic ischemic events. Exercise (4) and physical activity (5) may be associated with reductions in levels of inflammatory markers, including C-reactive protein (CRP) and interleukin-6 (IL-6), which could slow disease progression, functional decline, and reduce risks for cardiovascular outcomes in PAD patients (6–8). Additionally, inflammation may interfere with the protective functions of HDL (i.e., efflux capacity) (9–11). However, little is known about the association among HDL metrics associated with CVD risk, inflammation, and exercise in patients with PAD (12–14).

High-density lipoprotein structure (as measured by subfraction concentration) is associated with CVD risk (15, 16), and measurement of HDL function (as measured by HDL-C efflux capacity) has emerged as a robust marker of prevalent and incident CVD (17). The clinical significance and profile of HDL subfractions in patients with PAD are also not well understood with reports of both increased (18) and decreased (19) HDL3 levels in the setting of reduced HDL2 and HDL-C. The HDL subfractions, HDL2 and HDL3, are particles with unique size and density that may have distinct cholesterol efflux capacities (20), exhibit unique efflux responses to drug therapy (21), and have differential associations with CVD events (22). Thus, structural changes in HDL particles may be paralleled by functional changes with implications for CVD risk (23). A previous meta-analysis of exercise on HDL subfractions in adults without known CVD demonstrated that exercise is associated with increased HDL2 (1). However, the effect of supervised exercise on HDL subfraction concentration and HDL efflux capacity in patients with PAD has not been studied.

The study to improve leg circulation (SILC) was a 6-month randomized trial of supervised treadmill exercise, leg resistance training, or control in individuals with PAD with and without claudication that demonstrated improved physical function and quality of life in response to a supervised exercise intervention compared to control. First, in a *post hoc* analysis of the SILC study, we quantified the associations among HDL efflux, HDL subfractions, and circulating inflammatory markers at baseline in a cross-sectional analysis. Second, we evaluated the effect of 24 weeks of supervised exercise on changes in HDL efflux, HDL subfractions, and circulating inflammatory markers using changes in 6-min walk as a measure of response to the exercise intervention.

## Materials and Methods

### Design Overview

The SILC trial enrolled 156 patients with PAD with and without intermittent claudication between April 1, 2004, and August 8, 2008. Participants were recruited from newspaper and radio advertisements (*n* = 85), non-invasive vascular laboratories at Northwestern Memorial Hospital and other Chicago-area hospitals, and *via* community mailings and posters. The institutional review boards of the participating hospitals and medical centers approved the protocol. Patients were randomly assigned to supervised treadmill exercise (*n* = 51), lower extremity resistance training (*n* = 52), or a control (*n* = 53) group. Details of the design, patients, outcome definitions, and results have been published (24). Baseline information concerning demographics [age, sex, race, body mass index, ankle brachial index (ABI), and smoking], past medical history (leg symptoms, diabetes, angina, myocardial infarction, heart failure, and cancer), and statin and glitazone use were collected at the time of enrollment in SILC.

### Study Interventions

The supervised treadmill exercise intervention consisted of treadmill exercise three times a week for 24 weeks, supervised by an exercise physiologist. The duration and intensity of treadmill exercise was over the course of 24 weeks and/or at near maximal leg symptoms (if present). Participants in the lower extremity resistance training group exercised three times a week for 24 weeks with a certified trainer. They performed three sets of eight repetitions of knee extension, leg press, and leg curl exercises. For each exercise, one repetition maximum was measured at baseline and every 4 weeks, and weights were increased until participants lifted 80% of their one repetition maximum. The control group received 11 nutritional information sessions over 6 months. “Nutritional information sessions were not provided to the supervised exercise groups.”

### Leg Symptoms

Leg symptoms were assessed at baseline and follow-up using the San Diego claudication questionnaire (25). Intermittent claudication was defined as exertional calf pain that does not begin at rest, causes the subject to stop walking, and resolves within 10 min of rest (26).

### Six-Minute Walk

At baseline and follow-up, 6-min walk performance was assessed using standardized protocols (27). Participants walked up and down a 100-ft hallway for 6 min after instructions to cover as much distance as possible. The distance completed after 6 min was recorded.

### Ankle Brachial Index

A handheld Doppler probe (Nicolet Vascular Pocket Dop II; Nicolet Biomedical Inc., Golden, CO, USA) was used to obtain systolic pressures in the right and left brachial, dorsalis pedis, and posterior tibial arteries. Each pressure was measured twice. The ABI was calculated by dividing the mean of the dorsalis pedis and posterior tibial pressures in each leg by the mean of the four brachial pressures (28). Average pressures in the arm with the highest pressure were used when the first brachial pressure was higher than the opposite brachial pressure in both measurement sets and the second brachial pressures differed by 10 mm Hg or higher in the first measurement set (29).

### Circulating Inflammatory Markers

Interleukin-6 was measured using an ultrasensitive enzyme-linked immunosorbent assay (R&D Systems, Minneapolis, MN, USA). Concentrations of high-sensitivity CRP were determined using an immunoturbidimetric assay on the Hitachi 911 analyzer (Roche Diagnostics, Indianapolis, IN, USA), using reagents and calibrators from Denka Seiken (Niigata, Japan).

### Measurement of HDL-C Efflux Capacity

Two hundred microliters of stored serum (100 μl baseline and 100 μl follow-up) from PAD patients randomized to supervised exercise training and control were assayed for HDL-C efflux capacity.

Cholesterol efflux measurements were performed using the J774 radiolabeled cholesterol assay, which has been extensively used in studies of cholesterol efflux to human serum (30). J774 cells (ATCC, Rockville, MD, USA), derived from a murine macrophage cell line, are plated and radiolabeled with 2 μCi of 3H-cholesterol per milliliter. ATP-binding cassette A1 (ABCA1) is a transmembrane protein involved in reverse cholesterol transport (RCT) by functioning as a cholesterol efflux pump. ABCA1 is upregulated by means of an 18-h incubation with 0.3 mM 8-(4-chlorophenylthio)-cyclic AMP. Subsequently, efflux mediums containing 2.8% apolipoprotein B (apo-B)-depleted serum were added for 4 h. Apo-B-depleted serum was created by mixing 40 parts 20% PEG 6000 (QIAGEN Science, Germantown, MD, USA) with 100 parts serum with gentle mixing, and then incubated at room temperature for 20 min. Apo-B-depleted serum was then obtained by recovery of supernatant following centrifugation (10,000 rpm, 30 min, 4°C). All steps were performed in the presence of the acyl-coenzyme A:cholesterol acyltransferase inhibitor CP113,818 (2 μg/ml). This assay measures efflux mediated by several macrophage-specific RCT pathways, including the ABCA1- and ATP-binding cassette G1- (ABCG1) mediated transport, scavenger receptor B1-mediated transport, and aqueous diffusion (30, 31). Stimulation of J774 cells with cAMP upregulates ABCA1-mediated efflux. Thus in the conditions used here, total release of cholesterol occurred mainly by ABCA1 and passive diffusion.

Liquid scintillation counting was used to quantify the efflux of radioactive cholesterol from the cells. The quantity of radioactive cholesterol incorporated into cellular lipids was calculated by means of isopropanol extraction of control wells not exposed to patient serum. After serial washing with PBS, cell lipids were extracted from control culture dishes with isopropanol for 1 h, then evaporated to dryness under nitrogen gas, and were then reconstituted in chloroform. All assays were performed in triplicate. Percent efflux was calculated by the following formula:

$$\begin{array}{l} {}[(\text{microcuries of 3H - cholesterol in mediums containing 2.8\% apolipoprotein B-depleted}\\ \text{serum − microcuries of 3H - cholesterol in serum - free mediums})/\text{microcuries of 3H -}\\ \text{cholesterol in cells extracted before the efflux step}]\times 100. \end{array}$$

Values were normalized by dividing the efflux capacity of individual patients by the efflux capacity of a serum pool run with each assay.

### Lipoprotein and Subfraction Measurement

The vertical auto profile (VAP) test was used to obtain a comprehensive measurement of the lipoprotein cholesterol profile. One hundred forty-two microliters of stored plasma (71 μl at baseline and 71 μl post-exercise) were analyzed from PAD patients randomized to supervised treadmill training (*n* = 33), strength training (*n* = 29), and age- and gender-matched controls (*n* = 26). The VAP test is based on a well-established method of ultracentrifugation for lipoprotein subfraction determination (32). VAP provides cholesterol concentrations of various lipoproteins subclasses, including the HDL subfractions HDL2 and HDL3.

### Statistical Analysis

Baseline characteristics were summarized as means and SDs, frequencies, and percentages, as appropriate. Chi-squared tests and one-way analyses of variance were used to compare baseline characteristics of participants across the three groups. Two sample, two-sided *t*-tests were used to compare changes in outcomes between 6-month follow-up and baseline between each exercise group and the control group, respectively, without adjustments for multiple comparisons. The Shapiro–Wilk test was used to assess for normality of the data.

Relationships between continuous variables of interest were assessed using Pearson correlation coefficients (denoted as *r*) and multivariable adjusted linear regression models adjusted for age, race, gender, HDL subfractions, inflammatory markers, smoking status, statin, and glitazone.

Two sample, two-sided *t*-tests were used to compare changes in outcomes between baseline and 6-month follow-up among the treadmill, strength training, and control groups without adjustment for baseline data.

The *p* value considered statistically significant was *p* ≤ 0.05. All analyses were performed using the SAS statistical software (version 9.4, SAS Institute Inc., Cary, NC, USA).

### Sample Size Power Calculation

Since we used macrophage-to-plasma efflux capacity as in the study by Olchawa et al., sample size estimation was performed using the effect size and SDs of their study. To detect a difference of 2.6% (18.8 vs. 16.2%), we estimated that we would need 22 subjects per group to have an 80% chance of having a statistically significant result at *p* = 0.05.

## Results

### Study Participants

One hundred fifty-six PAD patients were enrolled in the SILC study, 68 were excluded because of lack of availability of baseline or follow-up serum sample. Patients were randomly assigned to supervised treadmill exercise (*n* = 51), lower extremity resistance training (*n* = 52), or a control (*n* = 53) group. Among those not included, there was a higher prevalence of heart failure (12 vs. 7%, *p* = 0.02) and cancer (17 vs. 11%, *p* = 0.04) compared to the subjects included in the current sub-study. The characteristics of SILC trial participants are shown in Table 1. The mean age of the participants was 70.85 ± 1.72 years. A total of 53.5% of patients were women and 46.5% were black. There were no significant differences in baseline demographics, CV risk factors or disease, severity of PAD, 6-min walk, or HDL-related parameters in any of the study groups. There were also no significant differences in baseline characteristics from study onset to completion following the study interventions.

**Table 1:** **Baseline characteristics of peripheral artery disease according to study group assignment**.

Baseline measures	Treadmill (*N* = 33)	Strength (*N* = 29)	Control (*N* = 26)	*p* Value
Age (years), mean (SD)	71.45 (8.58)	73.48 (8.58)	67.62 (11.68)	0.08
Ankle brachial index, mean (SD)	0.60 (0.18)	0.60 (0.14)	0.59 (0.18)	0.9
Male sex, *n* (%)	14 (42.42)	13 (44.83)	14 (53.85)	0.7
Black race, *n* (%)	16 (48.48)	7 (24.14)	13 (50.00)	0.08
Body mass index (kg/m^2^), mean (SD)	29.85 (5.84)	29.44 (7.08)	30.51 (8.46)	0.9
6-min walk distance (m), mean (SD)	337.26 (86.59)	306.03 (95.77)	325.42 (99.26)	0.4
Diabetes, *n* (%)	11 (33.33)	12 (41.38)	12 (46.15)	0.6
Current smoker, *n* (%)	8 (24.24)	5 (17.24)	10 (38.46)	0.2
Angina, *n* (%)	3 (9.09)	5 (18.52)	2 (7.69)	0.4
Myocardial infarction, *n* (%)	7 (21.88)	7 (25.00)	3 (11.54)	0.4
Heart failure, *n* (%)	2 (6.25)	3 (10.34)	2 (7.69)	0.8
Cancer, *n* (%)	6 (18.18)	3 (10.34)	2 (7.69)	0.4
HDL2-C (mg/dl)	13.79 (8.78)	11.76 (5.31)	12.65 (7.53)	0.6
HDL3-C (mg/dl)	38.09 (10.06)	33.83 (6.53)	34.65 (9.79)	0.1
HDL-C (mg/dl)	51.88 (17.99)	45.59 (10.70)	47.31 (16.62)	0.3
Normalized efflux	1.15 (0.20)	1.15 (0.20)	1.11 (0.25)	0.8
Intermittent claudication, *n* (%)	9 (27.27)	3 (10.34)	4 (15.38)	0.2
On statins, *n* (%)	21 (63.64)	17 (58.62)	13 (50.00)	0.6
On glitazones, *n* (%)	4 (12.12)	2 (6.90)	1 (3.85)	0.6

*HDL-C, high-density lipoprotein cholesterol; HDL2, high-density lipoprotein-2 cholesterol; HDL3, high-density lipoprotein-3 cholesterol*.

### Association of Baseline HDL Subfractions, Inflammatory Markers, Comorbidities, and Medications with Baseline Cholesterol Efflux Capacity

In all study patients, HDL-C (β = 0.006, *p* = 0.0001), HLD2 (β = 0.008, *p* = 0.0105), HDL3 (β = 0.011, *p* < 0.0001), and IL-6 (β = −0.019, *p* = 0.0294) but not CRP was associated with baseline HDL-C efflux capacity. However, after adjustment for age, race, gender, current smoking status, IL-6, statin, and glitazone use, only HDL3 (β = 0.0147, *p* = 0.0014) was associated with baseline cholesterol efflux capacity. Including diabetes, heart failure, coronary artery disease, and cancer into the adjusted model did not change the magnitude or statistical significance of the association between baseline HDL3 with baseline HDL efflux.

Additionally, there was no significant difference in baseline HDL-C efflux capacity in patients receiving statins vs. not receiving statins (mean efflux = 1.18 vs. 1.10, *p* = 0.61, respectively), or in patients receiving glitazones vs. those not receiving glitazones (mean efflux = 1.13 vs. 1.18, *p* = 0.21, respectively).

### Association of Baseline HDL Subfractions and Inflammatory Markers

**Figure 1:**
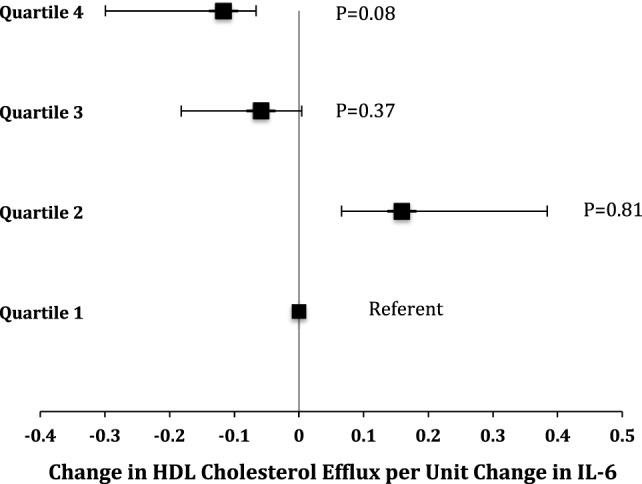
**Beta coefficients of linear regression modeling examining the association of baseline cholesterol efflux stratified according to baseline quartiles of interleukin-6 (IL-6) in all study participants**.

In an unadjusted analysis, we observed an inverse association between baseline IL-6 levels and baseline HDL3 concentration (β = −0.99, *p* = 0.008). This association was minimally altered (β = −0.94, *p* = 0.01) after adjustment for age, race, gender, current smoking status, comorbidities, statin, and glitazone use. In addition, among patients with the highest quartile of IL-6, a trend toward an inverse association was observed between baseline IL-6 and baseline efflux capacity (β = −0.1167, *p* = 0.08) (Figure 1).

### Association of Exercise with Changes in HDL Subfractions, Cholesterol Efflux Capacity, and Inflammatory Markers

**Figure 2:**
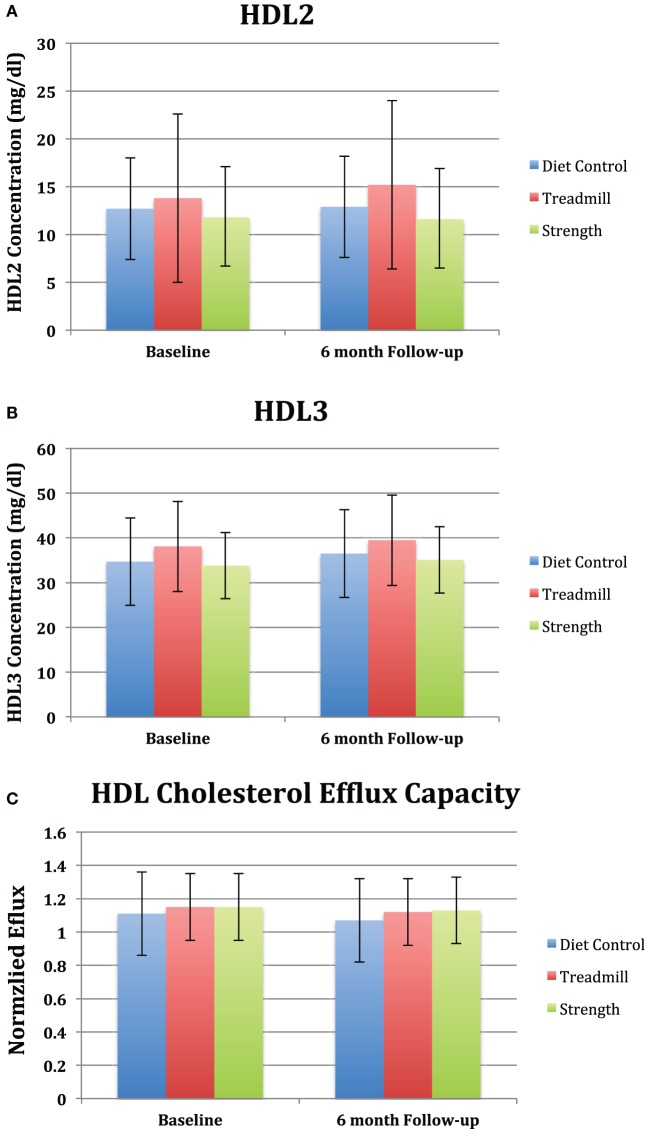

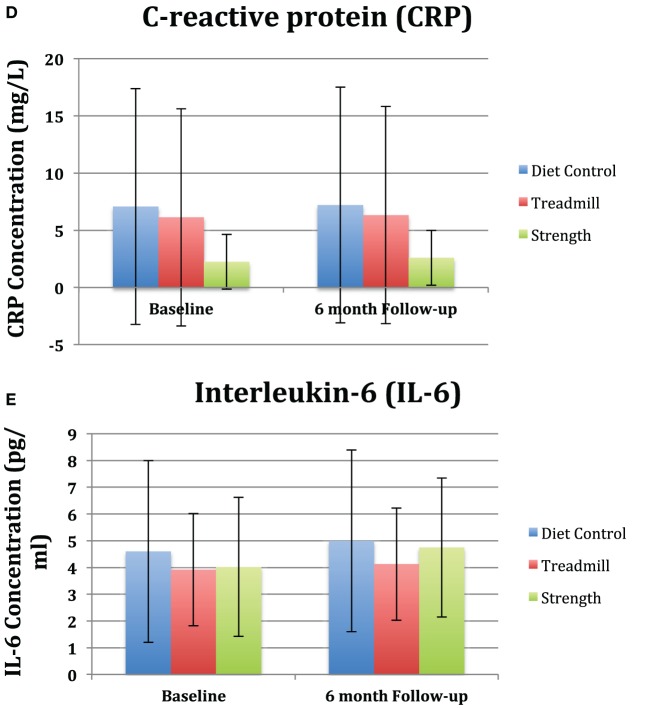
**Changes in (A) high-density lipoprotein (HDL) subfractions, (B) cholesterol efflux capacity, and (C) inflammatory markers among peripheral artery disease in study to improve leg circulation patients according to group assignment**. All values are average ± SE. **(A)** *p* = 0.38 for strength, treadmill, and diet control groups compared to baseline. For differences compared to control at 6-month follow-up: strength (*p* = 0.7) and treadmill (*p* = 0.3). **(B)** *p* = 0.9 for strength, treadmill, and diet control compared to baseline. For differences compared to control at 6-month follow-up: *p* = 0.7 for strength and *p* = 0.8 for treadmill. **(C)** *p* = 0.9 for strength, treadmill, and diet control compared to baseline. For differences compared to control at 6-month follow-up, *p* = 0.7 for strength and *p* = 0.7 for treadmill. **(D)** *p* = 0.99 for strength, treadmill, and diet control compared to baseline. For differences compared to control at 6-month follow-up, *p* = 0.9 for strength and *p* = 0.98 for treadmill. **(E)** *p* = 0.8 for strength, treadmill, and diet control compared to baseline. For differences compared to control at 6-month follow-up, *p* = 0.7 for strength and *p* = 0.8 for treadmill.

There were no significant changes in HDL2 and HDL3 compared to baseline in the strength trained group [(−0.17 mg/dl, 95% CI: −1.88 to −1.54) and (1.24 mg/dl, 95% CI: −0.85 to 3.33), respectively] and treadmill group [(1.42 mg/dl, 95% CI: −0.18 to 3.03) and (1.42 mg/dl, 95% CI: −0.54 to 3.39), respectively] (Figures 2A,B). Additionally, there was no significant difference in HDL2 and HDL3 in the strength and treadmill groups at 6 months follow compared to control.

No significant changes were observed in normalized cholesterol efflux capacity compared to baseline and control at 6-month follow-up in the strength trained group [(−0.02, 95% CI: −0.1 to 0.07) and (0.03, 95% CI: −0.1 to 0.15; *p* = 0.7), respectively] and treadmill group [(−0.02, 95% CI: 0.1–0.06) and (0.02, 95% CI: −0.1 to 0.14; *p* = 0.7), respectively] (Figure 2C). Additionally, no changes were observed in the mean levels of IL-6 or CRP from baseline to follow-up (Figures 2D,E). Lastly, there was no association between changes in HDL-C efflux capacity, HDL2, HDL3, or inflammatory markers (Table 2) among the treadmill, resistance trained, or control groups.

**Table 2:** **Association of changes in high-density lipoprotein (HDL) efflux, HDL subfractions, and circulating inflammatory markers with changes in 6-min walk**.

Change in	Pearson correlation coefficients		
	Change in 6-min walk		
	Treadmill	Strength training	Control
Efflux	0.0351 (*p* value = 0.8464, *n* = 33)	0.0729 (*p* value = 0.7070, *n* = 29)	0.0591 (*p* value = 0.7743, *n* = 26)
HDL2-C	0.1398 (*p* value = 0.4377, *n* = 33)	−0.3508 (*p* value = 0.0621, *n* = 29)	0.1049 (*p* value = 0.6099, *n* = 26)
HDL3-C	−0.0328 (*p* value = 0.8562, *n* = 33)	−0.2323 (*p* value = 0.2253, *n* = 29)	−0.0006 (*p* value = 0.9976, *n* = 26)
CRP	−0.0754 (*p* value = 0.6920, *n* = 30)	−0.0934 (*p* value = 0.6299, *n* = 29)	0.1432 (*p* value = 0.4947, *n* = 25)
IL-6	−0.3256 (*p* value = 0.0791, *n* = 30)	−0.2054 (*p* value = 0.2851, *n* = 29)	−0.1098 (*p* value = 0.6015, *n* = 25)

*Efflux, HDL-C efflux capacity; HDL2, high-density lipoprotein-2 cholesterol; HDL3, high-density lipoprotein-3 cholesterol; CRP, C-reactive protein, IL-6, interleukin-6*.

**Figure 3:**
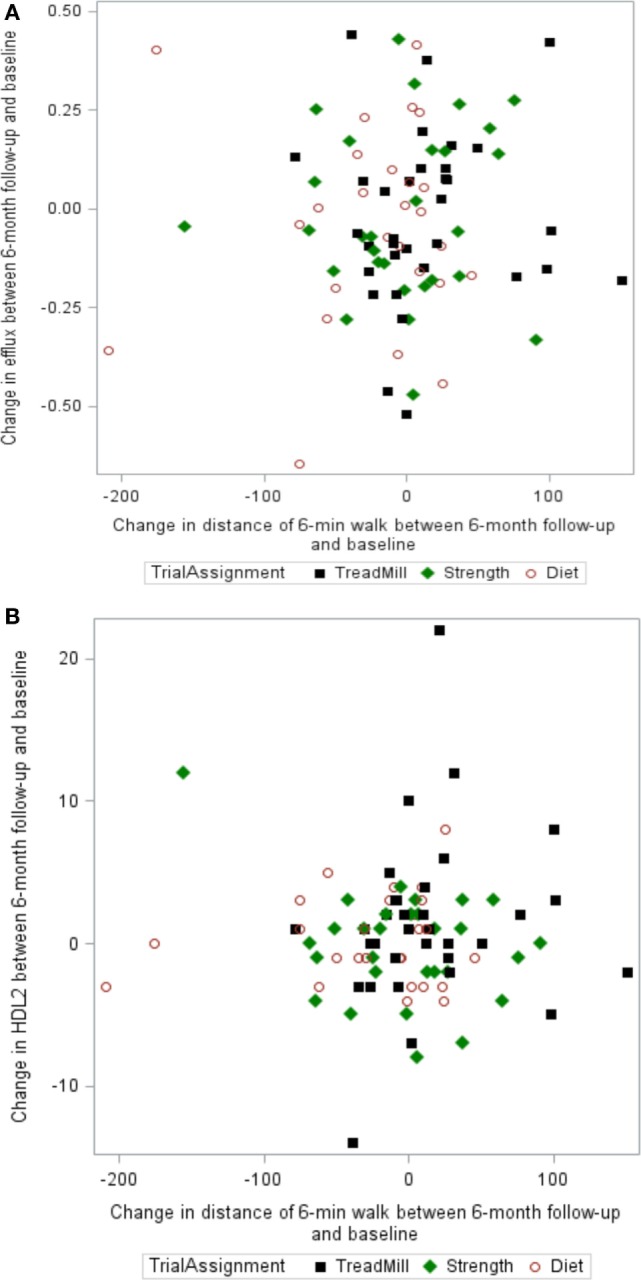

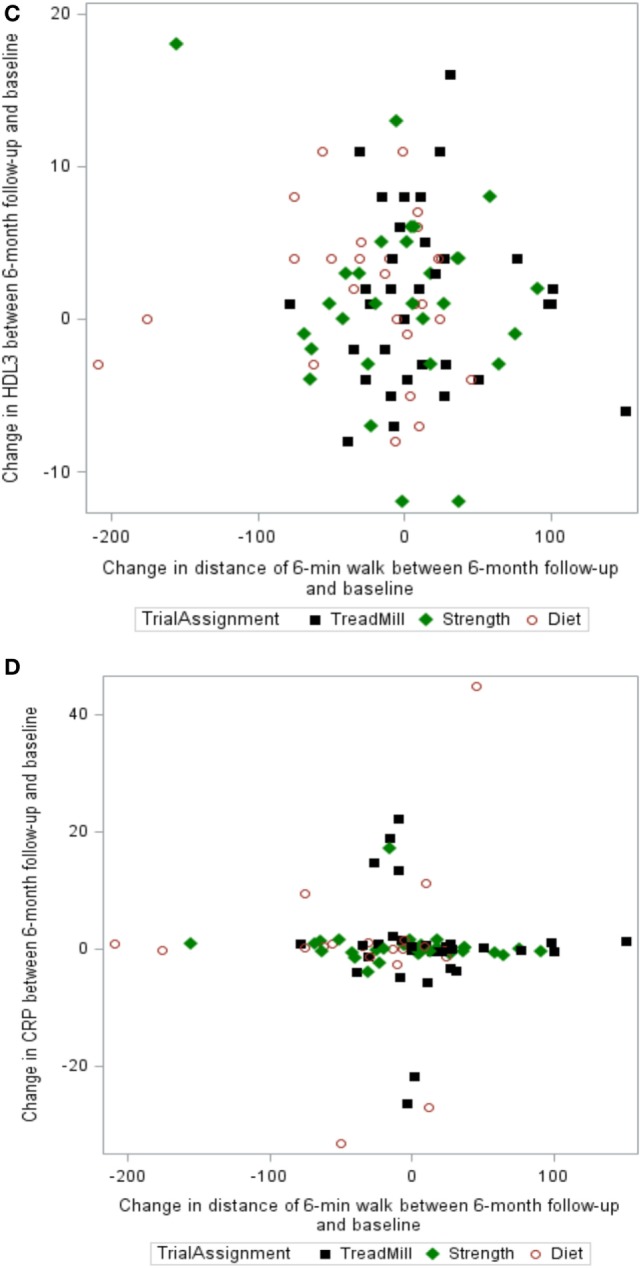

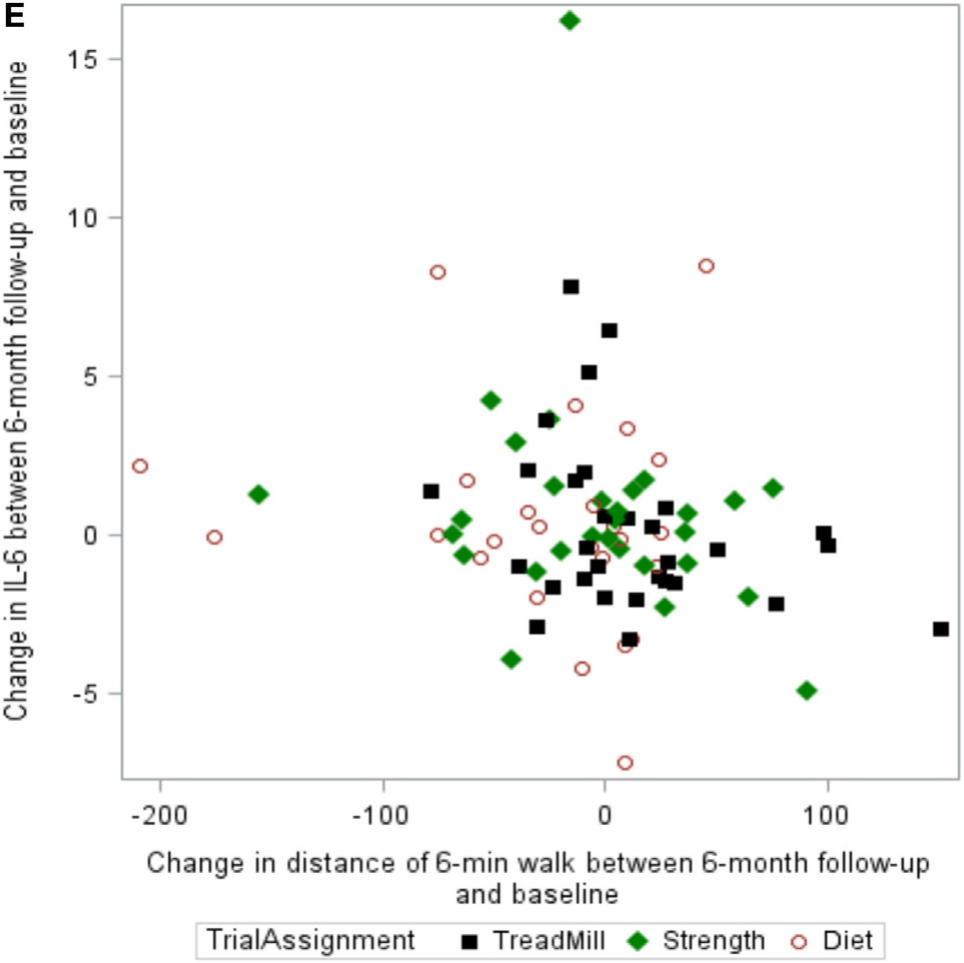
**Scatter plots of the relationship between changes in 6-min walk and changes in (A) cholesterol efflux capacity, (B) HDL2, (C) HDL3, (D) C-reactive protein (CRP), and (E) interleukin-6 (IL-6)**.

The relationship between changes in 6-min walk distance and changes in cholesterol efflux capacity, HDL2, HDL3, CRP, and IL-6 is shown for individual study participants in Figure 3. As depicted in Figure 3, there is no evidence of a linear relationship between changes in functional capacity as a result of the exercise interventions, HDL-related parameters, and inflammatory mediators.

## Discussion

Our results show that supervised strength and treadmill exercise training are not associated with changes in HDL subfractions, cholesterol efflux capacity, or inflammatory markers in patients with PAD. We observed an association between baseline HDL subfraction concentrations and HDL efflux, an inverse association between baseline IL-6 and HDL efflux, and an inverse association between baseline IL-6 and HDL3 concentration.

The SILC study demonstrated that exercise is associated with beneficial effects in patients with PAD, including improving walking distance, endothelial function, and quality of life (24). However, the mechanisms underlying the beneficial effects of exercise are not well understood in PAD. We studied an assay of HDL-mediated RCT thought to reflect the most relevant pathways involved in atheroprotection (33), and measured HDL subfractions that have been implicated in RCT (34, 35) to explore the biologic mechanisms underlying the favorable effects of exercise. Our results suggest that the beneficial effects of exercise in patients with PAD do not include favorable changes in HDL structure or function as measured by HDL subfraction or efflux, respectively. To our knowledge, this study represents the first investigation of the effect of structured exercise interventions on these HDL-related metrics in patients with PAD.

Supervised exercise did not alter HDL-C, structure, or function as measured in our *post hoc* analysis of the SILC cohort. A possible explanation for these findings is that a sufficient “exercise dose” may be necessary to increase HDL-C concentration in individuals without known CVD (1, 36, 37), and PAD patients may not exercise at a sufficient intensity to achieve such beneficial changes. Importantly, it is not known if such an exercise threshold exists to favorably remodel HDL subfractions and/or improve HDL efflux in patients with PAD, or even if HDL efflux is modified by exercise in patients with PAD. Koba et al. recently observed an increase in HDL efflux capacity compared to baseline following 6 months of cardiac rehabilitation and intensive lifestyle modification counseling in 57 patients with a recent acute coronary syndrome compared to baseline levels but no significant increase compared to 11 control patients who did not undergo these interventions (38). In a bivariate analysis, they observed that increased HDL efflux was associated with increased HDL-C, apolipoprotein A1, and high-intensity statin use. Interestingly, only patients who achieved a high level of exercise tolerance (defined as a peak VO_2_ ≥ 19 ml/min/kg) and “complete risk factor control” were observed to have a significant increase in HDL efflux capacity suggesting an exercise dose–response relationship may exist between exercise and efflux. In contrast to the PAD population examined in the current study, the study population of Koba et al. was significantly younger with fewer comorbidities and received a more intensive study intervention that included 6 months of supervised exercise and lifestyle modification counseling. Importantly, HDL-C may not increase with exercise in patients with baseline low levels of HDL-C (39) as is often observed in patients with PAD (40). Furthermore, increased skeletal muscle blood during physical activity and subsequent activation of lipoprotein lipase may be an important mechanism for increasing the concentration of HDL subfractions following exercise, a process that may be significantly diminished in patients with PAD and flow-limiting atherosclerotic lesions in the arterial circulation of their lower extremities (41).

Another possible explanation for our findings may be that patients with severe established atherosclerosis, such as those with PAD, have dysfunctional HDL that is not amenable to improvement *via* structured exercise or pharmacologic intervention (9, 10, 42). Contrary to other studies of cholesterol efflux capacity in patients with CVD, we did not observe a positive association between HDL efflux and treatment with statin or glitazone medications (30, 38, 43). We did observe, however, a univariate association between baseline IL-6 levels and HDL-C efflux capacity (which was lost after adjustment), and a multivariate association between HDL3 and efflux. PAD patients have elevated levels of IL-6 (44), which may modulate cholesterol efflux to HDL (45–47). It remains unclear if examination of a larger number of inflammatory mediators would have provided additional evidence of an association between inflammation and the HDL-related metrics examined in our study. We also observed that HDL-C efflux in the PAD subjects was elevated compared to the referent pool (i.e., normalized value above 1), and that HDL efflux was refractory to exercise intervention. A normalized efflux value greater than 1 suggests that HDL-C efflux capacity may have been near maximal and therefore not amenable to augmentation in this older cohort with advanced PAD (or that normalization of efflux was performed using an unhealthy referent pool).

Importantly, compared to other studies of HDL efflux and CVD, our study cohort was significantly older with more advanced atherosclerotic burden, and therefore exercise and pharmacologic interventions may be less effective in modifying efflux capacity than they might be in younger individuals.

Our study has several limitations. First, the analyses are *post hoc* and should be considered exploratory. Second, the small sample size may have contributed to lack of statistical power to detect an effect of exercise on efflux and HDL subfractions; however, our *a priori* power analysis suggested this was not so. Third, we examined many comparisons, and our analyses did not adjust for multiple comparisons. Our findings regarding baseline HDL subfraction, IL-6, and HDL efflux may be due to chance, and adjustment for multiple comparisons was not possible since most results of this study were not significant. Additionally, a number of factors may have affected the accuracy of this sample size estimate, including that the impact of chronic exercise has not been evaluated in patients with PAD and may be increased or decreased compared to healthy controls, which would either decrease or increase, respectively, the sample size requirements. In addition, Olchawa et al. study did not normalize efflux values relative to a control serum pool run with each assay. Using this normalization technique may theoretically increase the SDs and thus sample size.

## Conclusion

In this *post hoc* analysis of PAD patients from the SILC study, metrics of HDL structure (HDL3) and function (efflux) were inversely associated with a marker of systemic inflammation (IL-6) at baseline; however, the association of IL-6 with HDL efflux was lost after adjustment for comorbidities and HDL3. Supervised exercise did not alter the HDL subfraction profile, improve HDL-C efflux capacity, or modulate levels of CRP and IL-6 in PAD patients from SILC. Future studies of the effect of structured exercise on these HDL-related metrics may be warranted in patients with PAD and elevated inflammatory markers.

## Ethics Statement

Northwestern University Feinberg School of Medicine Institutional Review Board approved this study. Patient consent was obtained at the time of the original SILC study for study sample analysis.

## Author Contributions

Conceived study, designed experiments, and composed manuscript (MA, JW, and RM); financial support (JW and RM); edited manuscript (MA, ZW, YG, RM, and JW); and performed experiments and analyzed data (MA, ZW, and YG).

## Conflict of Interest Statement

The authors declare that the research was conducted in the absence of any commercial or financial relationships that could be construed as a potential conflict of interest.

## Abbreviations

HDL2, high-density lipoprotein-2 cholesterol; HDL3, high-density lipoprotein-3 cholesterol.
